# Impact of community programming targeting newcomer youth settlement and mental wellbeing: a scoping review

**DOI:** 10.3389/fpubh.2026.1882597

**Published:** 2026-08-07

**Authors:** Matthew Y. W. Kwan, Maggie Mclean, Ellen M. Wade, Taylor Rowe, Jayden Miller, Sujane Kandasamy

**Affiliations:** 1 Department of Child and Youth Studies, Brock University, St. Catharines, ON, Canada; 2 Department of Community Health Sciences, University of Calgary, Calgary, AB, Canada; 3 School of Human and Nutrition Sciences, University of Queensland, Saint Lucia, QLD, Australia; 4 Department of Health Sciences, Brock University, St. Catharines, ON, Canada; 5 Mary Heersink School of Global Health and Social Medicine, McMaster University, Hamilton, ON, Canada

**Keywords:** community programs, mental health and wellbeing, newcomers, settlement, youths

## Abstract

Newcomer youth represent a growing share of recent immigrants to Canada and often face substantial challenges as they navigate unfamiliar social, educational, and cultural environments. Community-based programs—including arts, sport, mentoring, and school-based supports—are increasingly recognized as important mechanisms for fostering resilience, belonging, and mental wellbeing. This review was initially registered as a systematic review; however, substantial heterogeneity in program types, study designs, and outcome measures necessitated a shift to a scoping review approach following PRISMA-ScR guidelines. Searches of PsycINFO, PubMed/MEDLINE, and Web of Science [2000–2025] identified 1,680 records, of which 17 studies met inclusion criteria. Programs varied widely, spanning creative arts therapies, peer and mentor-based interventions, school-based mental health supports, nutrition education, and multi-tier therapeutic models. Thirteen studies reported improvements in at least one domain of mental health and wellbeing, social connection, or settlement integration, highlighting benefits such as enhanced emotional expression, resilience, self-esteem, and school belonging. A smaller number of studies identified potential risks, including re-traumatization and discomfort with disclosure, underscoring the importance of culturally safe and trauma-informed delivery. Overall, the review demonstrates promising, but limited, evidence supporting community programming for newcomer youth and highlights a need for more rigorous, longitudinal, and community-engaged evaluations to guide policy and practice.

## Introduction

Canada is one Western nation that continues to experience high levels of newcomer arrivals, despite reduced immigration targets in the coming years. Among those arriving is a growing number of young newcomers—defined in this review as immigrant, refugee, and dependents of temporary foreign workers who have recently migrated to Canada—whom now represent approximately one in 10 recent immigrants (1, 2). This demographic shift underscores the urgent need for inclusive systems that can support newcomers youths (typically defined as the period between childhood and adulthood between the ages of 15 and 24 years) as they progress through a critical period of social, emotional, and developmental transition. Importantly, newcomer youths represent a heterogeneous population, but one that faces some unique challenges as they navigate unfamiliar cultural, educational, and social landscapes. Experiences may vary according to migration pathway, age, gender, settlement context and more, all of which shape their needs, experiences, and responses to community-based programming.

During the settlement process, newcomer youth are particularly vulnerable as they adapt to unfamiliar environments while managing the emotional and psychological stressors associated with migration. Many face intersecting challenges—such as trauma, cultural dislocation, precarious housing, discrimination, and systemic exclusion—that undermine their sense of wellbeing and belonging (3, 4). In practice, this often includes navigating complex education systems, overcoming language barriers, coping with disrupted schooling, and struggling to access or even become aware of available supports.

The settlement sector, particularly agencies tasked with supporting newcomer families, has been stretched thin in recent years. Aggressive immigration targets, combined with limited and uneven resource allocation, have left many organizations overextended (5, 6). While there is growing recognition that community engagement through sport, recreation, arts, and other leisure programs can play a critical role in promoting settlement and integration for newcomer children, youth, and their families, the reality is that many agencies lack the capacity and infrastructure to develop, deliver, or sustain such programming. Even where programs exist, organizations often struggle to map or coordinate the broader landscape of community offerings, resulting in fragmented and inconsistent access for youth across different regions (7, 8).

Despite these constraints, a range of culturally responsive programs has emerged across Canada, spanning mental health supports, mentorship initiatives, peer leadership training, and arts- and sport-based interventions. These programs have demonstrated promise in fostering integration, building resilience, and enhancing physical, mental, and social wellbeing (9–11). For example, sport- and recreation-based initiatives have been shown to facilitate social connections, reduce isolation, and provide safe spaces for newcomer youth to build skills and confidence (12, 13). Arts- and culture-based interventions have similarly been linked to improved self-expression, identity formation, and belonging (14, 15).

Mental wellbeing, broadly understood as a multidimensional construct encompassing mental, physical, and social domains, is particularly salient for newcomer youth. Research demonstrates that these domains are interrelated and mutually reinforcing (16). For young newcomers, wellbeing is shaped not only by individual health but also by their access to culturally safe environments, meaningful social networks, and opportunities for self-expression and development (14, 17). Programs that integrate these elements are thought to play a protective role in supporting settlement and integration. However, the literature examining such programming remains fragmented and inconsistently evaluated. Programs differ significantly in structure, delivery methods, and intended outcomes, making it difficult to draw broad conclusions or identify which approaches are most effective for specific subgroups of youth (18). While isolated studies provide evidence of positive impacts, including reductions in depressive symptoms, enhanced social connection, and smoother integration trajectories, the field lacks a comprehensive synthesis of findings. This limits our understanding of both the promise and limitations of community-based interventions in supporting newcomer youth.

The present review was initially conceived and registered as a systematic review, with the goal of synthesizing the impact of community programs on newcomer youth mental health and wellbeing or settlement outcomes. As the review progressed, the literature was found to encompass a wide range of program types, intervention approaches, and outcome domains. Accordingly, the purpose of this review is to synthesize existing literature on community programming aimed at newcomer youths and to examine how different types of interventions, program foci, and delivery modalities are associated with outcomes in three interconnected domains: (1) mental health and wellbeing, (2) social connection, and (3) settlement integration.

## Methods

### Study design and protocol

This review was conducted in accordance with the *Preferred Reporting Items for Systematic Reviews and Meta-Analyses extension for Scoping Reviews* [PRISMA-ScR; (38)], which served as a reporting guideline for the conduct and presentation of the review. The review was initially registered with the International Prospective Register of Systematic Reviews (PROSPERO). However, during the screening and data extraction stages, it became evident that the included literature encompassed a wide range of program types, study designs and outcome measures. This level of heterogeneity limited the feasibility of applying traditional systematic review evidence synthesis approaches and reduced the meaningfulness of direct comparison across studies. Thus, a scoping review approach was adopted *post-hoc* to capture the breadth of available literature, synthesize diverse findings, and identify gaps for future research. By mapping this landscape, the review identifies common patterns, highlights knowledge gaps, and points toward opportunities for future research and program development that can more effectively support newcomer youth across Canada.

### Eligibility criteria

Eligible studies included peer-reviewed articles published in English from January 2000 onward that examined programming for newcomer youth aged 15 to 24 years. Studies involving participants of all genders, ethnicities, and socioeconomic backgrounds were included. Any type of program was considered eligible, including extracurricular community-based, sport, mental health, arts, and mentorship initiatives, provided the program engaged directly with newcomer youth. Studies were excluded if the intervention targeted only adults, a focus on children under the age of 12, or newcomer populations without directly involving youth in the programming.

### Search strategy

Three electronic databases were searched: PsycINFO, PubMed, MEDLINE, and Web of Science. The search was conducted in the summer of 2025 and was restricted to English-language, peer-reviewed publications from January 2000 to August 2025. The search strategy combined key terms across four conceptual domains—youth, newcomer, wellbeing, and programming—using Boolean operators. Searches were limited to English-language, peer-reviewed publications published from 2000 onward. Specifically, terms for youth (e.g., “youth,” “adolescents,” “teen”) were combined with newcomer-related terms (e.g., “newcomer,” “refugee,” “immigrant,” “migrant”), wellbeing terms (e.g., “wellbeing,” “mental health,” “sense of belonging,” “integration”), and program-related terms (e.g., “program,” “intervention,” “leisure,” “recreation,” “sport,” “physical activity,” “after-school”). The final search query required articles to include at least one term from each domain.

### Study selection and screening process

**Figure 1:**
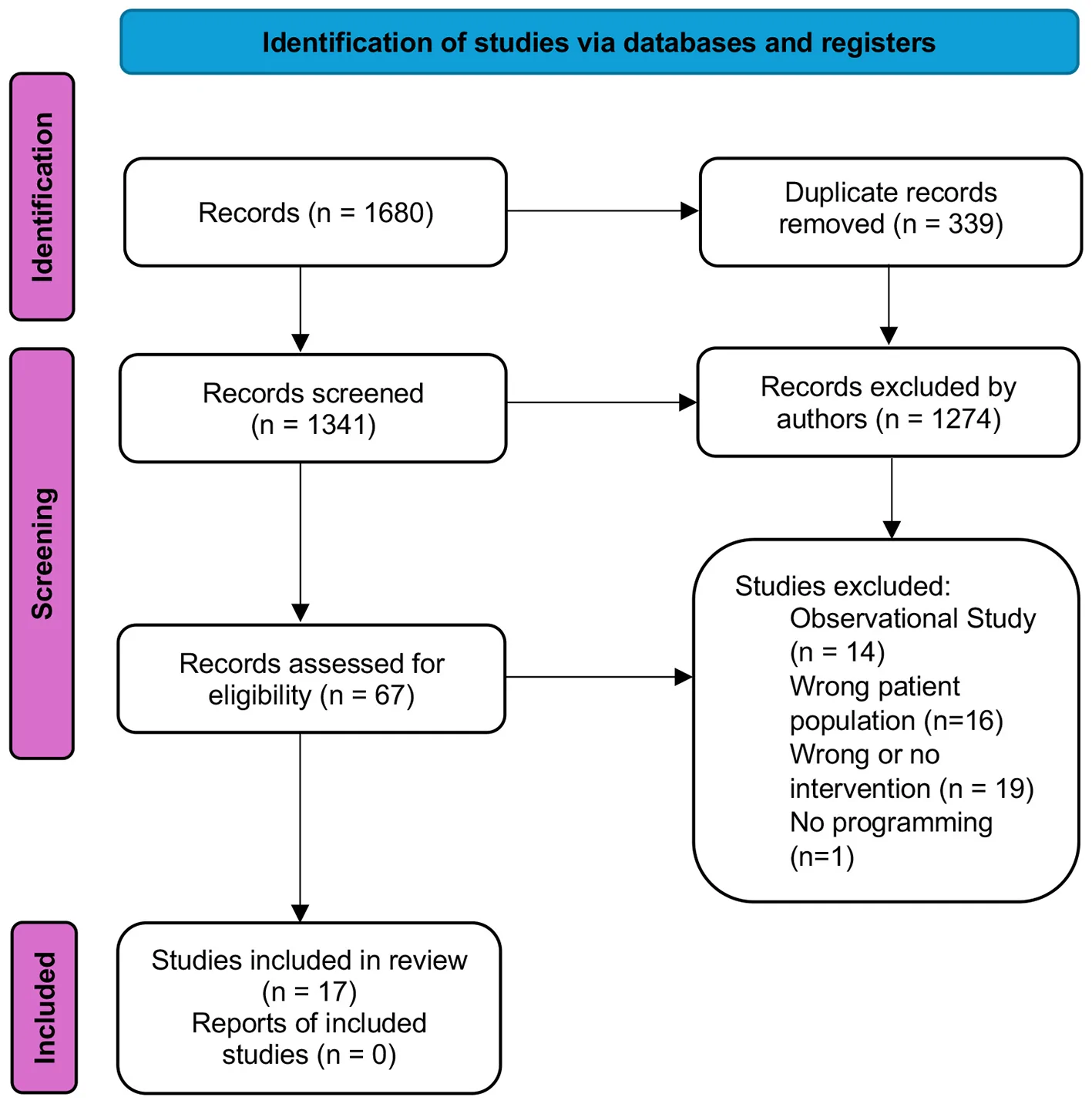
Consort diagram of included studies.

Two reviewers independently screened all titles and abstracts in Covidence following duplicate removal. A pilot screening of 10 abstracts was undertaken to refine inclusion criteria. Articles with two “Yes” votes advanced to full-text review; those with two “No” votes were excluded. Discrepancies (e.g., Yes/No, Yes/Maybe) were resolved through team discussion until consensus was reached. Our initial search yielded a total of 1,680 studies, including 339 duplicates that were identified by the software and subsequently removed. The remaining 1,341 studies were screened based on title and abstract against the inclusion criteria. During this stage, 60 articles required discussion to resolve screening conflicts between reviewers. A total of 67 studies proceeded to full-text screening, of which 17 met the study eligibility criteria and were included in the final synthesis (see Figure 1).

### Data extraction

Data from included studies were charted using a standardized extraction template developed in Covidence to ensure consistency and transparency across the review process. Extracted information captured multiple domains: (1) program characteristics (e.g., type of intervention, duration, setting, and delivery modality); (2) participant demographics (e.g., age, gender, country of origin, and time since arrival); (3) study design and methodology (qualitative, quantitative, or mixed-methods); (4) reported outcomes, categorized into the three core domains of mental health and wellbeing, social connection, and settlement integration; and (5) contextual factors such as publication year, study location, and author information. Ambiguous or missing information, as well as conflicts, were flagged during extraction and were resolved through discussion. The structured template facilitated comparison across studies and enabled identification of patterns in program design, focus, and outcome measurement.

### Data synthesis

Given the heterogeneity of program types, study designs, and outcome measures, a narrative synthesis was employed. Quantitative findings were summarized descriptively, while qualitative findings were synthesized using thematic analysis. Following data extraction, study findings were reviewed and coded according to program characteristics, intervention approaches, and reported outcomes. Codes were iteratively grouped into broader categories based on patterns and commonalities across studies. Through an inductive process, thematic categories were developed to capture recurring program features and outcomes and were also informed by existing frameworks on youth development, migration, and integration [e.g., (5, 14)]. Emerging themes and categories were discussed among members of the research team and refined through an iterative review process to ensure consistency and interpretive rigor. This approach enabled the review to capture both the breadth of program models and the mechanisms through which programming may influence newcomer youth outcomes.

## Results

A total of 17 studies met the inclusion criteria, representing a wide range of methodological approaches and geographic contexts (see Table 1). Designs included mixed-methods case studies and quasi-experiments (*n* = 5), evaluation studies (*n* = 3), qualitative research (*n* = 4), and quantitative experimental or quasi-experimental designs (*n* = 5). The majority of studies were conducted in Canada and the United States; additional evidence emerged from Europe, Australia and Asia. Collectively, these studies highlight a heterogeneous but growing body of research exploring community-based and arts- or mentoring-focused interventions aimed at supporting newcomer youth's wellbeing, social connectedness, and integration.

**Table 1:** Programs supporting settlement and wellbeing in newcomer youth (*n* = 17).

No	Authors (Country)	Study design & participants	Program activity	Program target	Results
1	Smith and Crooks (31) (Canada)	Mix-methods case study Immigrants, refugees, other, and unknown (*n* = 95)	Healthy Relationships Plus–Enhanced: 8 weekly online group sessions (~1 h); facilitated by staff (1–15 yrs experience with newcomer youth); focus on discussions & interactive activities around healthy relationships.	Investigate the acceptability of an evidence-informed healthy relationships promotion and violence prevention program for newcomer youth.	Youth highly endorsed the program, especially sessions on communication, boundaries, stress, and peer pressure. They emphasized the usefulness of these skills for navigating social interactions and applying them in relationships, and also valued the trust and cohesion built over time.
2	Seff et al. (24) (Detroit)	Quasi-experimental pilot evaluation Refugee and immigrant (*n* = 108)	Forward with Peers: 10-week program within school, weekly ~1 h sessions in three high schools; led by a bilingual English/Arabic facilitator; focused on culturally adapted SEL and life skills (mental health, cultural awareness, bullying, leadership, financial literacy, computer skills, career exploration, goal-setting); used trauma-informed and culturally sensitive approaches.	Explore the feasibility and potential effectiveness of an integrated social emotional learning (SEL) and life skills program, Forward with Peers.	All outcomes of interest, except hope, improved or remained stable in the treatment group, while they worsened or remained stable in the control group. Participants in the treatment arm reported significantly greater increases in overall perceived social support and support from a “special person,” with marginal gains in resilience and family support. Positive trends were also observed in reduced loneliness and suicide ideation, as well as improved school belonging.
3	Smith et al. (30) (United Kingdom of Britain and Northern Ireland)	Qualitative study Refugees (*n* = 6)	3-year Participatory Action Research with BelongHere (London charity); 11 semi-structured stakeholder interviews; youth-led photovoice with 6 young leaders (ages 17–19, refugee/UASC status); participant observation and reflexive practice; framework analysis.	Examine how young people from refugee backgrounds understand their capabilities, the ways they negotiate and contest those capabilities, and the potential negative unintended consequences for their wellbeing within community sport and leisure contexts.	The program supported youth capabilities, particularly by fostering affiliations, attachments, and opportunities for control and bodily integrity. Risks included re-traumatization and reinforcement of unequal power hierarchies through tokenistic involvement in decision-making. Successes were linked to attention to holistic wellbeing, secure relationships, protection from harm, and genuine youth participation in decision-making around sport and leisure.
4	Kim et al. (20) (Republic of Korea)	Quasi-experimental design Immigrants (*n* = 24)	Art Therapy for Korean Youth: 5 group sessions (~90 min each); facilitated by qualified art therapists; used structured art therapy techniques to support emotional expression, stress relief, and coping strategies.	Examine the effects of an art therapy program on psychological outcomes, acceptability, and satisfaction among Korean immigrant youth.	Youth expressed consistently positive views of the arty therapy sessions, with improvements in emotional expression, reduced stress, and early signs of stronger coping strategies. Structured art therapy showed benefits for wellbeing and adaptation among immigrant youth.
5	Garcia et al. (26) (USA)	Evaluative study Immigrant (*n* = 51)	Immigrant Youth Program (IYP): Full-day alternative high school for Central American newcomer youth; integrates English immersion, TASC (GED) prep, and career training; led by bilingual staff and social workers; fosters cultural inclusion, relational safety, and belonging to support academic and personal growth.	Evaluate IYP, a full-day alternative high school program, to promote immigrant students' wellbeing, cultural integration, and academic success.	Students reported gains in community and belonging, motivation from faculty, teacher flexibility, support services, cultural safety, and feeling understood. They emphasized trust in teachers and valued efforts to understand their experiences. The IYP approach supported bicultural identity development, encouraging students to honor their identities while engaging with American values and culture.
6	Pryce et al. (23) (Canada)	Quasi-experimental Immigrant (*n* = 92)	Conversation Club (CC): After-school group mentoring program (Fall 2013–Spring 2014) across six sites (high schools, community centers, libraries); facilitated through Big Brothers Big Sisters; focus on ethnic identity, belonging, and wellbeing.	Examine the effects of the CC program on Canadian newcomer adolescent experiences of belonging, hopefulness, connectedness, and integration into Canadian society.	Youth expressed that the program created a safe space to connect with peers, share experiences, and strengthen their sense of identity and belonging. They reported feeling more comfortable in their schools and communities, noting that discussions and mentoring helped them build confidence and form friendships. The program was associated with reduced feelings of isolation and fewer depressive symptoms, as participants highlighted the value of having supportive mentors and relatable peers.
7	Quinlan et al. (21) (Australia)	Quasi-experimental design Refugee (*n* = 42)	HEAL creative arts therapy: A group intervention delivered to newcomer students in an intensive English language stream. Sessions used creative arts therapy techniques to provide a supportive space for self-expression, cultural adjustment, and emotional wellbeing within the school environment.	Controlled trial testing creative arts therapy to address psychosocial needs of refugee-background students.	The HEAL creative arts therapy program improved emotional symptoms, with moderate effect sizes for reducing behavioral difficulties and peer problems. Therapists noted that newly arrived refugee youth often found it difficult to reflect on therapy while in high school, becoming more open to sharing their experiences after transitioning to mainstream schools.
8	Kowitt et al. (37) (USA)	Non-randomized post-test pilot Refugee (*n* = 9)	Pilot art therapy program for refugee youth from Burma, delivered through individual and group therapy sessions by trained therapists (average 5.5 years in the U.S.); sessions emphasized creative expression as a tool for processing trauma and supporting adjustment.	Showed how art therapy organizations can integrate program evaluation into practice using existing frameworks, engaging stakeholders, and strengthening organizational capacities.	Data revealed high mental health burden, with depression and anxiety rates above national averages. The pilot showed evaluation activities were feasible, client-responsive, and realistic, while also building organizational capacity.
9	Ferrera et al. (27) (USA)	Qualitative study Immigration status was not directly assessed; however, participants were identified as youth from immigrant-origin backgrounds (*n* = 23)	Youth empowerment program in Chicago known as the Young Health Service Corps (YHSC). The program trained participants in community health and leadership skills, combining education with hands-on service projects to foster empowerment, civic engagement, and health knowledge.	To analyze Chicago's Youth Health Service Corps through a CBPR lens—examining it as a health promotion program, exploring CBPR in immigrant communities, and discussing preliminary findings using a critical youth empowerment model.	Youth experienced the program as empowering at both individual and community levels. Benefits included strengthened social capital (leadership skills, college and healthcare preparedness), capacity building, and health outreach through their community networks.
10	Ellis et al. (19) (USA)	Qualitative study Refugee (*n* = 30)	Project SHIFA: A multi-tier mental health program implemented in a U.S. middle school with Somali refugee youth. The program focused on Tier 2 support, which involved school-based resilience-building groups for Somali students and served as a screening pathway for more intensive supports.	To establish that refugee youths who receive a multi-tiered approach to services, Project SHIFA, would show high levels of engagement in treatment appropriate to their level of mental health distress, improvements in mental health symptoms, and a decrease in resource hardships.	Students across all program tiers showed mental health improvements, with no differences between Tier 2, 3, and 4 services—indicating the multi-tiered model provided students with the appropriate level of care they needed.
11	Rousseau et al. (33) (Canada)	Cluster randomized control trial Permanent resident, refugee/asylum seeker, other (*n* = 55)	Pilot intervention delivered to under-schooled adolescent refugees and immigrants in Canada. Conducted in classrooms, the program combined drama and language-based creative expression to support psychosocial adjustment.	Presents the results of a pilot study evaluating a intervention of drama workshops integrating activities in multiple languages.	Youth used the workshops to share stories of loss and trauma, supported by the protective group setting. They expressed pride in using their own or others' languages as recognition of their backgrounds, which facilitated self-expression. Some withdrew when asked to use their native language or discuss their country, with more positive outcomes when given freedom to choose language and topics.
12	Castro-Olivio and Merrell (32) (USA)	Pre-post intervention design Immigrant (*n* = 40)	*Jóvenes Fuertes* (Strong Teens – culturally adapted SEL program): 12 weekly classroom lessons (~45–50 min) led by bilingual, bicultural teachers; focused on topics such as emotional awareness, stress and anger management, empathy, positive thinking, problem solving, and goal setting; incorporated Latino cultural values (e.g., respeto, familismo), folktales, and content on acculturative stress and ethnic pride	Describe the process of adapting an evidence-based SEL programme for use with a group of Latino immigrant adolescents enrolled in public schools in the USA.	Vast majority of participants reported being highly satisfied with the program, would recommend the program to other students, and saw themselves using the presented skills in their lives.
13	Rousseau et al. (28) (Canada)	Randomized controlled trial Permanent resident, refugee, other (*n* = 123)	Classroom Drama Therapy program implemented in integration classes at a multiethnic high school in Canada. The intervention was based on Augusto Boal's forum theater techniques and used drama activities to help immigrant and refugee adolescents express emotions, build social connections, and support psychosocial adjustment. The program was delivered during school hours and compared against control classes without drama therapy.	The goal of the drama therapy program was to give young immigrants and refugees a chance to reappropriate and share group stories, in order to support the construction of meaning and identity in their personal stories and establish a bridge between the past and present	Drama workshops did not have direct effects on symptoms reported by teachers and adolescents, however it did reduce adolescents' perceived symptom-related impairment and interference with friendships, home life, and leisure. Effects varied by gender—reducing impairment for girls and preventing increases for boys. No measurable effect was found on self-esteem, though qualitative feedback indicated youth felt greater self-knowledge and wellbeing.
14	Rousseau et al. (29) (Canada)	Cluster randomized control trial Refugee and immigrant – 1st and 2nd generation *(n = 477)*	Classroom-based creative expression interventions delivered in public schools through two formats: Classroom Drama Workshops (theater-based activities) and Tutorship sessions. The interventions targeted refugee and immigrant adolescents to foster social integration, expression, and adjustment within the school setting.	Evaluate the effectiveness of a theater intervention program for immigrant and refugee youth in special classes for improving mental health and academic outcomes.	The theater intervention did not reduce self-reported impairment or symptoms more than tutoring or control conditions. Findings suggest drama workshops may aid adaptation for first-generation newcomers but risk reinforcing exclusion and perceptions of dysfunction among second-generation youth.
15	Dähne et al. (25) (Denmark)	Case study design Refugee and migrant (*n = 41)*	Classroom Drama Workshops (CDWs) delivered in seven public schools to adolescent refugees and. CDWs used role-play, storytelling, and drama-based activities to foster self-expression, peer interaction, and adaptation in the classroom setting.	Aimed to yield insights into the implementation process of Classroom Drama Workshops (CDWs) in Danish preparatory classes.	CDWs fostered enhanced sociability, stronger peer and student–teacher trust, and opportunities for fun and storytelling that promoted connection and mental health among participants. Some participants, however, reported discomfort with the level of personal sharing expected.
16	Alarcón et al. (22) (Barcelona)	Sequential explanatory mixed method design Migrant (*n* = 44)	Mentoring program delivered to migrant youth as part of the Referents programme; included structured mentoring sessions with trained mentors to build resilience, self-esteem, and psychological wellbeing.	This research examines the effects of participation in a mentoring programme on the psychological and educational outcomes among unac- companied migrant youths who live in the Barcelona metropolitan area.	Youth in the mentoring program reported higher self-esteem and resilience and lower psychological distress compared to controls. Participants emphasized the value of supportive mentor relationships and the opportunity to develop coping skills, highlighting mentoring as an effective strategy to promote psychosocial wellbeing in migrant youth.
17	Oliveira et al. (36) (Portugal)	Program development and evaluation Refugee (*n* = 15)	Nutritional education program developed and delivered at a refugee children's reception center (March–June 2016); consisted of educational sessions and practical activities to build knowledge of healthy eating.	Establish a nutritional and food education program to improve the integration process of young orphan refugees newly arrived in Portugal.	Participants showed gains in nutritional knowledge and increased awareness of healthy eating practices. The program highlighted the feasibility of integrating targeted nutritional education into reception center programming to support refugee youths' health and adjustment.

Across the included studies, intervention modalities varied considerably. Most were community-based mentoring and group programs, although some community programs run within schools were included. Several programs used creative expression modalities—notably drama, photovoice, and art therapy—designed to provide safe spaces for cultural expression, emotional regulation, and identity building. Other approaches included nutrition education within reception centers and full-day alternative high school models integrating academic and psychosocial supports. Delivery was generally group-based, though some models incorporated individualized mentoring or tiered levels of care [e.g., (19)].

The type of study design also shaped the insights generated. Mixed-methods studies typically captured both outcomes and implementation processes, illustrating how programs fostered resilience, coping skills, and identity development while also identifying challenges in sustaining effects over time. Evaluation studies primarily focused on feasibility, highlighting improvements in social support, resilience, and belonging, while also noting organizational capacity gains. Qualitative work provided youth perspectives, emphasizing empowerment and community building, but also revealing unintended harms such as tokenistic participation or re-traumatization. Quantitative studies, often programs delivered at schools, provided controlled evidence of modest improvements in emotional symptoms, coping skills, and social belonging, though several showed null or mixed effects depending on program design, gender, or migration status. Where multiple publications arose from the same intervention, each publication was retained because it reported distinct study designs, follow-up periods, participant samples, or outcomes. During synthesis, findings were interpreted collectively to avoid over-representing evidence from a single intervention.

The majority of the included studies found positive outcomes for newcomer youth, with 13 studies reporting improvements in at least one domain of mental health and wellbeing, social connection, or settlement integration. Programs commonly indicated that they found enhanced emotional expression (20, 21), self-esteem and resilience (22), and reduced isolation or depressive symptoms (19, 23). Interventions delivered within school often fostered greater belonging and peer trust (24, 25), while community and mentoring models promoted empowerment, bicultural identity, and leadership development (26, 27).

Four studies reported mixed or null findings: for example, drama workshops showed reductions in impairment but no consistent effects on self-esteem or symptoms (28, 29), and one art therapy program did not sustain all effects at follow-up (20). A small number (*n* = 3) highlighted potential negative or unintended consequences, including re-traumatization when youth were asked to revisit past experiences (30), discomfort with personal disclosure (25), or reinforcement of exclusionary dynamics in educational contexts (29). Overall results found most programs yielding meaningful psychosocial benefits, although careful attention is needed to implementation context, sustainability, and youth-centered safeguards to avoid unintended harm.

## Discussion

This scoping review mapped the evidence on community-based programs designed to support newcomer youth, with a focus on mental health and wellbeing, social connection, and settlement outcomes. The review revealed a small but growing body of work focusing on mental health and wellbeing and settlement outcomes among newcomer youth. Importantly, a systematic search of the literature identified significant heterogeneity in the study designs as well as the modality for which community interventions are being delivered—or studied. These included mentorship and peer support programs, arts-based interventions, nutrition education, and full-day alternative school models. Despite this diversity, programs appear most beneficial when they are culturally responsive, youth-centered, and embedded within supportive environments (21, 23, 24, 26, 31). Across the included studies, culturally responsive programming was operationalized through bilingual or bicultural facilitation, incorporation of participants' cultural identities and languages, trauma-informed delivery, opportunities for cultural expression, flexible adaptation to participant needs, and meaningful engagement of newcomer communities in program delivery. Collectively, these characteristics appeared to create psychologically safe environments in which newcomer youth could build trust, strengthen cultural identity, and develop meaningful connections with peers and mentors.

Across the included studies, the strongest and most consistent evidence related to improvements in mental health and psychological wellbeing. Programs employing mentoring, creative arts, social-emotional learning, and trauma-informed approaches frequently reported improvements in emotional expression, resilience, coping skills, self-esteem, and reductions in stress, depressive symptoms, or psychological distress (19–24). Notably, interventions that created psychologically safe environments and emphasized trusting relationships appeared particularly effective in supporting newcomer youth as they navigated the complex emotional challenges associated with migration and resettlement (31, 32). However, the findings also suggest that mental health gains are not universal. Several studies reported modest or mixed effects, particularly when programs relied on emotional disclosure or revisiting traumatic experiences, highlighting the importance of trauma-informed facilitation and participant autonomy (20, 25, 28–30, 33). Collectively, these findings reinforce growing evidence that community programming can serve as an important protective factor for newcomer youth mental health, particularly when interventions prioritize relational safety, flexibility, and culturally responsive delivery.

A second prominent finding was the consistent emphasis on strengthening social connection and fostering a sense of belonging. Across mentoring programs, classroom-based interventions, arts initiatives, and peer support models, youth frequently described developing stronger relationships with peers, mentors, teachers, and community members (23–26, 31). These relationships appeared to reduce feelings of isolation while promoting trust, social support, and confidence in navigating new environments (23, 24). Importantly, social connection emerged not simply as an outcome of programming but as a potential mechanism through which broader improvements in wellbeing were achieved. From the perspective of social capital theory, these programs appear to facilitate the development of bonding social capital through supportive peer relationships while also fostering bridging social capital by connecting newcomer youth with mentors, educators, and broader community networks that can facilitate access to information, opportunities, and resources (34). Likewise, the findings align with the concept of a greater sense of belonging as a fundamental human need (35), whereby opportunities to build meaningful relationships and participate in supportive environments enhance feelings of membership, belonging, and mutual support. Collectively, these findings suggest that fostering social connectedness represents one of the primary mechanisms through which community programming supports newcomer youth and promotes positive developmental outcomes.

While relatively few studies directly evaluated settlement trajectories, several reported improvements in indicators associated with successful integration, including stronger school engagement, bicultural identity development, leadership skills, civic participation, and increased confidence navigating educational and community systems (23, 26, 27, 36). Mentorship, school-based programming, and youth leadership initiatives appeared particularly well positioned to facilitate these broader integration processes by connecting youth with trusted adults, community resources, and opportunities to participate meaningfully within their new communities (22, 23, 26, 27). These findings are consistent with Ager and Strang's (5) Framework of Integration, which conceptualizes successful settlement as extending beyond access to services to include social connections, education, language, safety, rights, and a sense of belonging within the receiving community. The programs identified in this review appear to strengthen several of these domains, particularly social bridges and bonds, while also supporting educational engagement and community participation. However, relatively few studies explicitly evaluated longer-term settlement outcomes, such as educational attainment, employment readiness, civic participation, or sustained community engagement. Future evaluations should therefore incorporate broader indicators of settlement success to better understand the extent to which community programming contributes to successful integration over time.

Of note, although several interventions focused specifically on unaccompanied refugee or migrant youth, the available evidence was insufficient to determine whether different program models are required for accompanied and unaccompanied newcomer populations. Given their distinct settlement experiences and support needs, this represents an important priority for future research.

Despite the promising findings identified across this review, a substantial disconnect remains between the breadth of community programming being delivered and the limited evidence evaluating its effectiveness. Newcomer-focused programs are implemented daily across schools, settlement agencies, governments, and community organizations; however, relatively few have undergone rigorous evaluation. Consequently, the predominance of small-scale, pilot, and cross-sectional studies limits the ability to identify best practices, guide policy, or support sustainable implementation. Future research should therefore prioritize larger, longitudinal, and multi-site evaluations that assess both implementation processes and longer-term developmental and settlement outcomes.

### Implications for practice

Findings from this review reinforce the important role community-based programming can play in supporting newcomer youth. Across intervention types, programs appeared most effective when they were culturally responsive, trauma-informed, youth-centered, and relationally grounded. Collectively, the findings suggest that investing in these core principles may strengthen newcomer youths' mental health, social connectedness, and settlement experiences while supporting more equitable access to community participation.

### Implications for research

This review highlights a substantial gap between the widespread delivery of community-based programs for newcomer youth and the limited number that have been empirically evaluated. Despite clear promise across diverse modalities, the predominance of small-scale, short-term, and methodologically heterogeneous studies limits the field's ability to draw robust conclusions about effectiveness, mechanisms, and equity of impact. Future research should prioritize rigorous yet practice-embedded evaluation designs, including longitudinal, mixed-methods, and multi-site studies that can capture both outcomes and implementation processes in real-world settings. Advancing this work will require stronger and more equitable community–academic partnerships, as community organizations often lack the infrastructure or resources to conduct formal evaluations independently. Co-designed research approaches can help ensure that evaluation questions, methods, and outcomes are meaningful to youth and community stakeholders, while also strengthening the ecological validity and translational value of findings. Meaningful co-design should involve newcomer youth and community partners in shaping research priorities, informing outcome selection, and developing practice recommendations. This approach may enhance cultural relevance, responsiveness, and the practical utility of research findings. Greater attention to implementation factors—such as fidelity, adaptation, cultural safety, and organizational context—will also be essential to inform sustainable scaling of effective programs.

### Study limitations

While this review represents one of the first efforts to synthesize evidence on newcomer-focused community programs supporting settlement, several limitations warrant consideration. First, consistent with scoping review methodology, a formal assessment of study quality was not conducted. Therefore, the findings provide an overview of the existing literature and should not be interpreted as definitive evidence of program effectiveness. Second, the review was limited to peer-reviewed, English-language publications, potentially excluding relevant gray literature, community reports, and evaluations not disseminated through academic outlets. Because many newcomer programs are delivered by community organizations with limited research capacity, the breadth of programming in practice is likely underrepresented. Third, substantial heterogeneity in study designs, program modalities, and outcome measures limited comparability and precluded conclusions about effectiveness. Many included studies were small-scale, pilot in nature, or lacked control groups, constraining the strength of causal inferences. Fourth, outcome measurement varied considerably, while some studies used validated instruments for mental health or social connection, others relied on qualitative accounts or descriptive data, complicating synthesis. Finally, publication bias remains a possibility, as studies with positive results are more likely to be published than those reporting null or negative outcomes.

## Conclusion

This scoping review synthesized a diverse but fragmented body of literature examining community programming for newcomer youth. Across the evidence, programs demonstrated the greatest promise for improving mental health and wellbeing, strengthening social connectedness, and supporting broader settlement and integration processes. While these findings highlight the potential value of community programming, they also reveal a critical need for more rigorous evaluation to better understand which approaches are most effective, for whom, and under what circumstances. Strengthening partnerships between researchers and community organizations will be essential to generating the evidence needed to support sustainable, culturally responsive programming for newcomer youth.
